# Modeling Evolution of Cutting Force in Ultrasonically Assisted Drilling of Carbon Fiber Reinforced Plastics

**DOI:** 10.3390/ma15093392

**Published:** 2022-05-09

**Authors:** Ci-Rong Huang, Bing-Mai Liao, Chen-Yu Kai, Cheng-Mu Su, Jui-Pin Hung

**Affiliations:** 1 Intelligent Machinery Technology Center, Industrial Technology Research Institute, Taichung 40852, Taiwan; cironghuang@itri.org.tw (C.-R.H.); cykai@itri.org.tw (C.-Y.K.); 2 Graduate Institute of Precision Manufacturing, National Chin-Yi University of Technology, Taichung 41130, Taiwan; s860303s@gmail.com (B.-M.L.); fe5757@gmail.com (C.-M.S.)

**Keywords:** ANOVA, carbon fiber reinforced plastics (CFRP), cutting force model, delamination, regression analysis, Taguchi method, ultrasonically assisted drilling (UAD)

## Abstract

In this study, the effects of process parameters (feed rate, spindle speed, and ultrasonic power level) on the cutting force and delamination in the ultrasonic vibration-assisted drilling of carbon fiber-reinforced plastics (CFRPs) have been investigated. A series of drilling tests under various conditions defined by the design of experiment technique were conducted. The evolution of the cutting force during drilling cycles was measured and analyzed. Experimental analysis results based on the Taguchi method and analysis of variance show that the spindle speed is an influential factor affecting the cutting force with a contribution of 75.36%, and the feed rate significantly affects the delamination damage with a contribution of 46.57%. In addition, the cutting force was found to increase with drilling cycles at different rates, which depends on the process parameters used in drilling. The evolution behavior of cutting force was well fitted based on the process parameters by proposed regression models. Experimental validation indicates that the predicted forces show reasonable agreement with measured values under different conditions and reveal good prediction performances, with a root mean square error of 5.6 and a mean absolute percentage error of 5.8%. In drilling tests with variable cutting conditions, the evolution of the cutting forces predicted based on the selected parameters was successfully verified when compared with the measured results, with RMSE and MAPE values of 7.55 and 5.61%, respectively. As a conclusion, this predictive model provides an effective basis for selecting appropriate drilling parameters to suppress the cutting force on CFRP composites.

## 1. Introduction

Carbon-fiber-reinforced plastics (CFRPs) are widely used in aerospace, defense, military, automobile, and aircraft components owing to their excellent properties, such as high strength, high rigidity, low density, high temperature resistance, corrosion resistance, and fatigue resistance [1,2]. Drilling is one of the most typical processing methods in the application of carbon fiber composites. Carbon fiber composites are anisotropic and non-uniform; furthermore, they exhibit low bonding strength between layers and are significantly affected by temperature. Therefore, composite materials are susceptible to various mechanical damages during the drilling process, such as the delamination of laminated layers, pull-out fibers, unsatisfactory roundness or irregular surface of the inner wall, and severe tool wear, which severely affect their application in the aerospace industry [3,4].

The drilling mechanism shows that the composite plate exerts cutting forces at the entry and exit of the material, resulting in different delamination phenomena in the composite material [1,5]. These problems are associated closely with and significantly affected by the tool geometry features [6,7,8,9,10,11] and process parameters [12,13,14,15,16] used in the drilling process. For example, Abrao et al. [7] investigated the effects of four different geometric blade shapes on the drilling forces and material delamination of glass-fiber-reinforced epoxy composites. The results indicated no direct relationship between the drilling force and material delamination; however, it is significantly related to the geometry of the drill bit and the cutting parameters used in drilling. Frank [8] conducted a drilling experiment on long fiber-reinforced thermoplastics and discovered that the radius of the cutting edge of the auger bit significantly affected the machining results. A larger cutting edge radius generated a larger cutting force, which deteriorated the drilling quality. Meanwhile, Heisel et al. [10] conducted drilling experiments using drills with different drill apex angles and cutting parameters to observe their effects on the cutting force and drilling quality. They discovered that a larger drill top angle resulted in a better quality of the entry end; however, the quality of the outlet end was unsatisfactory. The drill top angle significantly affected the cutting force.

To realize the effects of drilling parameters, Zhang et al. [12] investigated the use of high-speed rigid twist drills in CFRP drilling and discovered spalling at the entry and deformation defects at the exit of the material, which became more severe as the feed rate increased and the spindle speed decreased. Krishnaraj et al. [13] discovered that neither the cutting speed nor feed rate resulted in significant delamination at the entry end of a test piece. Davim and Reis [14] confirmed that the feed rate affected the cutting force the most significantly, whereas the cutting speed slightly affected the axial cutting force. Geier and Szalay [15] conducted experiments using diamond-coated twist drills to analyze the effects of the cutting speed and feed rate on the delamination of CFRP specimens. Their experimental results confirmed that a higher feed rate increased the exit delamination, whereas cutting speed did not significantly affect the delamination at the exit; these were similar to the observations reported in [16,17,18,19]. As observed by Gaitonde et al. [16], high-speed cutting plays a major role in reducing damage at the entrance of the hole. In addition, the combination of low feed rate and point angle is also essential in minimizing delamination during the drilling of CFRP composites.

Furthermore, Hintze et al. [20] evaluated the total cutting force during unidirectional drilling and discovered that the components of the cutting force (cutting force, feed force, and passive force) were significantly affected by the cutting angle and that tool wear caused an increase in the cutting force. Hassan et al. [21] performed cutting experiments based on customized requirements. Subsequently, they proposed the optimal tool geometry (such as helical and point angles) and cutting parameters (spindle speed and feed rate) based on the maximum cutting forces measured in drilling as well as hole qualities.

When processing composite materials, the use of different processing parameters affected the cutting force and delamination damage to different extents. These effects can be more clearly assessed by using Taguchi’s experimental design and multiple regression analysis [22,23,24,25]. Enemuoh et al. [22] used the Taguchi method to develop multi-objective optimization techniques for drilling thermoplastic composite materials, including material delamination, surface roughness, and cutting force, to obtain an optimal solution that can avoid material delamination. Davim and Reis [14] used a design of experiments method to analyze the correlation among cutting parameters (cutting speed and feed rate), cutting power, and delamination factor. Tsao and Hocheng et al. [23] investigated the relationship among spindle speed, feed rate, drill diameter, and the delamination factor of CFRP laminates and then performed multiple regression analysis to establish a prediction model of the delamination factor for different drill bit types. Khashaba et al. [24] investigated the effects of cutting speed and feed rate on cutting thrust, cutting torque, and delamination factor. The empirical model thus established can be applied to select processing parameters for drilling composite materials with different fiber volume ratios.

Gaitonde et al. [16] applied a full factorial design to conduct a high-speed drilling of composite materials to investigate the effects of machining parameters on the delamination factor. They proposed a second-order nonlinear regression analysis model to analyze the effects of cutting speed, feed rate, and drill tip angle on sheet delamination. The results showed that the delamination trend decreases with increasing cutting speed, whereas the use of low-nose-angle tools and lower feed rates reduces machining damage. Shetty et al. [24] investigated the effects of cutting speed, feed rate, drill tip angle, and drill bit diameter on cutting force; subsequently, they combined a neural network model and a genetic algorithm to establish a cutting force prediction model and to perform process parameter optimization. Their final verification showed that the difference between the prediction efficiency of the neural network model and the regression model was less than 1%. It is obvious that drilling under different combinations of the process parameters will lead to different results in drilling quality and tool wear states. Basically, drilling at a higher cutting speed with a lower feed rate is well recognized to generate lower thrust forces and hence cause less damage to the composite laminate surface. Additionally, the interactive effects between the feed rate and cutting speed may show different influence extents on the drilling performances when the drill was designed with different geometry [9,10,11].

Rotary ultrasonic vibration-assisted machining has been widely used in the machining of difficult-to-machine materials [26,27,28,29,30]. Researchers who applied ultrasonic machining for drilling CFRPs confirmed that this technology can effectively reduce the drilling force and tool wear as well as improve the surface quality of the hole [28,30,31,32,33,34,35,36]. Sanda et al. [28] reported the difference between conventional and ultrasonic vibration-assisted drilling (UVD) methods for CFRP drilling; they confirmed that the cutting force generated by UVD can be reduced by 3% to 42%, and the delamination extent can be reduced significantly. Reductions in the cutting force and cutting torque greatly depend on the machining parameters used in drilling. Dahnel et al. [31] experimentally compared the effects of cutting speed on tool wear during drilling using conventional and ultrasonic vibration-assisted machining. The results revealed that ultrasonic-assisted machining can reduce tool wear and cutting force, and that was more advantageous than conventional machining methods. Ultrasonic vibration-assisted machining techniques are not only used in thermosetting resin composites but also in thermoplastic resin composites.

A more significant reduction in the cutting force and less effect on material delamination was achieved when lower feed rates were used in CFRP drilling [32,33,34]. Makhdum et al. [33,34] confirmed that ultrasonic vibration CFRP drilling can effectively reduce the degree of cutting-edge wear. The cutting force increases as the cutting edge wears out, but the increase in the cutting force in UVD continuous cutting cycles is significantly reduced as compared with conventional methods. Wu et al. [35] investigated the effects of processing parameters, such as feed rate, ultrasonic amplitude, and spindle speed, on the delamination of carbon fiber composite drilling and confirmed that ultrasonic vibration-assisted drilling can effectively suppress delamination damage when compared with conventional drilling. Geng et al. [36] processed small-diameter composite specimens using a rotatory ultrasonic helical machining module with a diamond grinding rod. The results showed that the drilling thrust decreased with an increase in the spindle speed, as well as with an increase in the helical and axial feed speeds. Compared with conventional drilling, this advanced method yields 57.8–71.3% lower cutting forces and 12.8–25.7% lower delamination factors. Recently, Cao et al. [37] proposed an analytical model to model drilling behavior and conducted drilling experiments using corn drills with abrasive diamond. Both approaches revealed that a higher feed rate increased the cutting force, whereas a higher spindle speed and ultrasonic vibration amplitude decreased the cutting force. In addition, the extent of material delamination was associated significantly with the cutting force. Therefore, the drilling force has a direct influence on drilling-induced damage in CFRP composites; hence, it is recognized to be the main parameter affecting the quality of a drilled hole.

The abovementioned studies reveal that, to improve the drilling performance of carbon fiber composite materials, the appropriate geometrical design tools should be used and the feed rate or cutting speed should be appropriately adjusted to reduce the cutting force and material delamination [38,39]. Tamura and Matsumura [39] proposed a variable feed rate method to increase the processing speed without causing material delamination. This further implies that the adjustment of the process parameters is important for maintaining the machining performance with better quality during the long-term machining process. In addition, as reviewed from the literature, most research works relating to the current study report the influences of process parameters on the cutting forces in the drilling process, attempting to find the appropriate parameters to reduce the cutting force and delaminating damages. But the evolution of drilling behavior with increasing drilling cycles such as variation in cutting force was not considered, which cannot provide an effective strategy to improve the drilling performance. The evolution of the drilling force during continuous drilling significantly affects the tool condition and hence the drilling performance and material quality, which should be discussed further. Additionally, in practical machining, monitoring the variation in the cutting force with the increasing drilling cycle can be effective for adjusting the process parameters and improving the cutting performance. Regarding this, a force sensing system is a perquisite for achieving the on-line monitoring of the machining quality and control of the process parameters. To be an affordable system, a predictive model of the cutting force can be regarded as an alternative tool worthy of development. Hence, based on this concept, the effects of different cutting conditions on the cutting force in the ultrasonically assisted drilling of CFRP were investigated in this study. The evolution of the cutting force with the drilling cycles under various cutting conditions was examined. Predictive models of the cutting force were established based on data acquired from drilling experiments under various cutting conditions. Finally, validation tests were conducted to demonstrate the application of the proposed model in the drilling process. The predictive model is expected to provide an effective basis for selecting appropriate drilling parameters to suppress the cutting force on CFRP composites.

## 2. Experimental Setup

### 2.1. Experimental Apparatus

**Figure 1:**
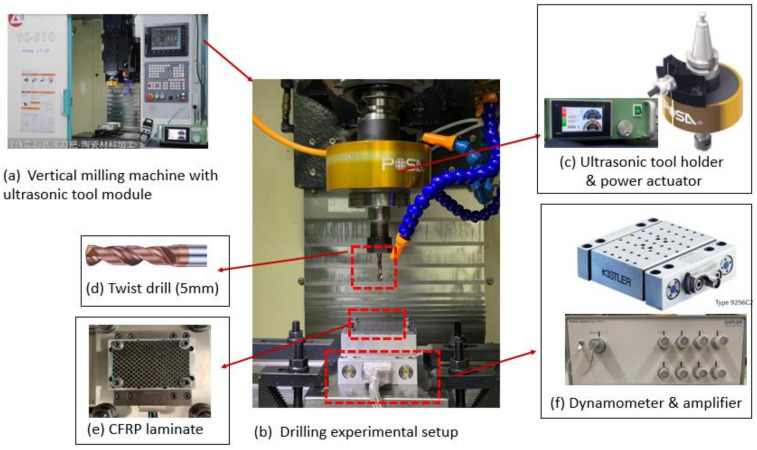
Experimental configuration: (**a**) vertical milling machine with ultrasonic tool module, (**b**) ultrasonic drilling experimental setup, (**c**) ultrasonic tool holder and power actuator, (**d**) twist drill, (**e**) CFRP workpiece, and (**f**) dynaometer with amplifier for measuring the cutting force.

In this study, drilling experiments of the CFRP workpiece were conducted on the vertical milling machine (Model TC510, L.K. Machinery Corp.; Taichung, Taiwan) with the ultrasonic vibration assisted milling module (Posa, Inc.; Taichung, Taiwan) and a data acquisition system for measuring the cutting force. The experimental setup is shown in Figure 1. The ultrasonic vibration module primarily comprises an ultrasonic tool holder and a wireless power supply. The ultrasonic tool holder can be inserted into the spindle nose, and the cutter can vibrate in the longitudinal direction at a high frequency of approximately 32 kHz via wireless power transmitters. The amplitude of the ultrasonic vibration can be adjusted under different settings of the output control of the power supply. The data acquisition system comprises a dynamometer (Model 9256C2, Kistler Inc.; Winterthur, Zürich, Switzerland), a charge amplifier (Model 5167A, Kistler Inc.; Winterthur, Zürich, Switzerland), and an A/D converter.

The CFRP laminates used in the drilling studies were provided by Yuan-Hsin Carbon Fiber Enterprise Co., Ltd., Miaoli, Taiwan, which were prepared from pre-impregnated carbon fiber layers in orientation 0°/90° with a fiber-epoxy matrix volume fraction of approximately 60%. The workpiece contained 10 layers of carbon fibers and had a size of 75 mm × 55 mm × 3 mm. The mechanical properties of the carbon fiber are as follows: Fiber diameter 7 μm, Filament 3000, Elastic modulus 230 GPa, Tensile strength 3530 MPa. The cutting tool was a tungsten carbide twist drill with a 140° drill-point angle and a diameter of 5.0 mm.

### 2.2. Design of Experiments

The process parameters used in this study, including the spindle speed (S), feed rate (F), and ultrasonic power rate (P), are shown in Table 1. The power rate of the ultrasonic excitation was used to control the vibration amplitude of the tool, which is an important factor affecting the machining performance of the UAD system. Each parameter was specified at three levels, which resulted in an orthogonal array L_9_ (3^3^) with nine different combinations of the process parameters. Nine drills were used under each specific cutting conditions. For each tool, the drilling operations were repeated for 25 cycles (25 holes) under the same cutting conditions. The cutting force in each drilling cycle was measured using a dynamometer mounted on the fixture of the table used. Thus, the evolution of the cutting forces with increase in the drilling cycles can be observed. In addition, the effects of the process parameters on the cutting force were investigated by means of statistical analysis based on the Taguchi technique. The Taguchi method was used to estimate the statistical performance, such as the signal-to-noise (S/N) ratio. The experimental results were evaluated by converting them into S/N ratios. In the S/N ratio, S is the signal factor and N is the noise factor. More specifically, S is the actual value obtained from the experiments, and N refers to factors that are excluded from the experimental design but affect the data in the investigation. The quality characteristic of the S/N ratio can be classified into three types: nominal-the-better, higher-the-better, and lower-the-better [40]. In this study, the minimum cutting force was desired; therefore, the smaller-the-better model was used and defined as follows [40]: Yi is the measured value of the cutting force for the ith drilling test under specific cutting conditions repeated in n trials.

(1) $$\frac{S}{N}=-10\log_{10}\left[\frac{1}{n}\sum_{i=1}^{n}Y_{i}^{2}\right]$$

### 2.3. Measurement of Cutting Force

**Figure 2:**
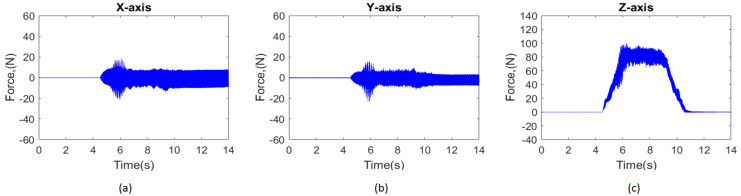
Typical time histories of cutting forces (*Fx*, *Fy,* and *Fz*) measured in the drilling process. (**a**) *X*-direction, (**b**) *Y*-direction, (**c**) *Z*-direction.

The CFRP specimen was fixed on a special fixture on the dynamometer, which was clamped on the machine table. The dynamometer (Model 9256C2, Kistler Inc.; Winterthur, Zürich, Switzerland) was connected to a personal computer with Dynoware software (Dynoware 2825D, Kistler Inc.; Winterthur, Zürich, Switzerland) to record the cutting forces from the amplifier (Model 5167A) during the drilling process. The raw data extracted from the software were then transformed to numerical data through MATLAB software. Figure 2 shows the typical time histories of the cutting forces (*Fx*, *Fy*, and *Fz*) measured in three axes (*X*-, *Y*-, and *Z*-directions) within a drilling cycle. As shown, the highest force occurs in the feeding direction (*Z*-axis), whereas the forces in the *X*- and *Y*-directions are significantly lower and hence not considered in the subsequent regression analysis. The average drilling force over the feeding period was used as a characteristic variable to assess the effects of the cutting conditions in this study.

### 2.4. Measurement of Ultrasonic Vibration Amplitude of Cutter

**Figure 3:**
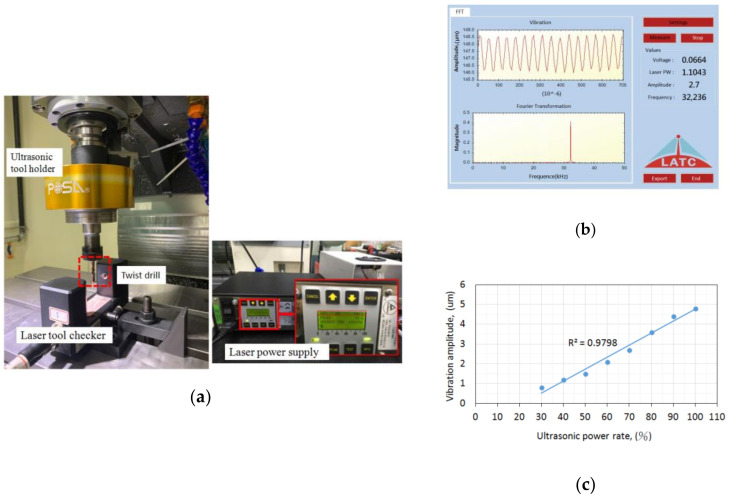
Measurement of the vibration amplitude of the ultrasonic tool holder. (**a**) Ultrasonic tool holder and tool checker with power supply, (**b**) graphic data display interface, (**c**) relationship between vibration amplitude and power rate.

In this experiment, a laser tool checker (Model UTC65, Latc corp., Taichung, Taiwan) was used to measure the vibration amplitude of the carbide tool, as shown in Figure 3a, in which a twist drill with diameter of 5 mm was inserted in the tool holder collet with an overhang length of 30 mm. The measurement was conducted under the stationery state of the spindle tool. The vibration amplitude was controlled by varying the output level of the power supply from 30% to 100%. At each power level, the laser tool checker automatically measures the vibration amplitude and frequency of the tool, as shown in Figure 3b, which indicates that the tool vibrates at 32 kHz with an amplitude of 2.7 μm under a power rate of 70%.

Figure 3c shows the relationship between the longitudinal amplitude of the ultrasonic vibration and the excitation power rate, which clearly shows that the vibration amplitude increases linearly with the power. The amplitudes of the tool are approximately 0.8 and 1.5 μm at power rates of 30% and 50%, respectively.

### 2.5. Measurement of Delamination

**Figure 4:**
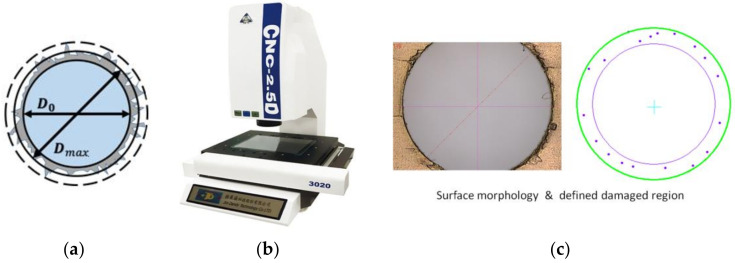
Quantification of the delamination of drilled CFRP. (**a**) Scheme of the delamination factor, (**b**) Optical vision measuring device, (**c**) Surface morphology and defined damaged region of the drilled hole.

During drilling, the interlaced laminated layers of the fiber material can easily rupture, tear, break, or delaminate because of the interaction between the laminated material and cutting edges under the drilling thrust force. Such damage to the CFRPs can be quantitatively characterized by the delamination factor (*F_a_*) and subsequently used to evaluate the effects of process parameters on drilling quality. In this study, *F_a_* is defined as the ratio of the maximum diameter around the damaged zone (*D_max_*) to the non-destructive inner hole diameter (*D*_0_) [14], as shown in Figure 4.

(2) $$F_{a}=\frac{D_{max}}{D_{0}}$$

The delamination zone of the drilled holes was measured using an automatic optical vision measuring machine with a resolution of 1.0 µm, as shown in Figure 4.

## 3. Multivariable Regression Analysis

### 3.1. Mathematical Function

Generally, a suitable approach for the functional relationship between the independent variables and dependent variables can be determined by using response surface methodology [41]. With this, a predictive model of the cutting force can be established based on the process parameters of an ultrasonic vibration-assisted machining system.

In the response surface methodology, all independent variables are correlated with the response in nonlinear form as [41]

(3) $$Y=\beta_{0}+\sum_{i=1}^{N}\beta_{i}X_{i}+\sum_{i=1}^{N}\beta_{\mathrm{ii}}X_{i}^{2}+\sum_{i}\sum_{j}\beta_{i}\beta_{j}X_{i}X_{j}+\varepsilon$$

where *Y* is the desired model of the dependent variable or the cutting force; *X_i_* and *X_j_* are the input variables or the cutting parameters; *β_i_*, *β_i__i_*, and *β_j_* are coefficients of the linear, quadratic, and interaction terms, respectively; and *i*, *j* = 0, 1, 2, …; ε is the total truncation error.

In this study, the mathematical function of the cutting force (*F_t_*) is assumed to be associated with the cutting parameters, i.e., the spindle speed (*S*), feed rate (*F*), and power rate (*P*) of ultrasonic excitation, by a nonlinear model in the following forms:

1. Nonlinear polynomial model

(4) $$F_{t}={\;\beta }_{0}+\beta_{1}N+\beta_{2}F+\beta_{3}S+\beta_{4}\cdot P+\beta_{5}\cdot F^{2}+\beta_{6}\cdot S^{2}+\beta_{7}\cdot F\cdot S+\beta_{8}\cdot N^{2}$$

2. Nonlinear power-law model

(5) $$F_{t}=\;C\cdot {F\;}^{\beta 1}\;\cdot {S\;}^{\beta 2}\cdot P^{\;\beta 3}\cdot N^{\;\beta 4}$$

This model can be expressed in logarithmic transformation form, as follows

(6) $${\ln F}_{t}=\;\ln C+\beta_{1}\cdot \ln F+\beta_{2}\cdot \ln S+\beta_{3}\cdot \ln P+\beta_{4}\cdot \ln N$$

In the models above, the regression coefficients *βi* (*i* = 0, 1, 2) are estimated from the experimental data through least-squares regression analysis.

### 3.2. Determination of the Effectiveness of Prediction Models

The prediction performance of the regression models was evaluated based on the root mean square error (RMSE), determination coefficient (R), and mean absolute percentage error (MAPE), which are defined as follows [42,43]:

(1) Root mean square error (RMSE)

Root mean square error represents the difference between the original and predicted values extracted by the root squared average difference over the dataset. It provides an indication regarding the dispersion or the variability of the prediction accuracy.

(7) $$RMSE={\left(\left(\frac{1}{N}\right)\left(\sum_{i=1}^{N}|t_{i}-y_{i}{|}^{2}\right)\right)}^{1/2}$$

(2) Determination coefficient (R)

The coefficient of determination measures the amount of variation accounted for by the fitted model. It can be interpreted as how well the predicted values fit compared with the measured values.

(8) $$R^{2}=1-\left(\sum_{i=1}^{N}(t_{i}-y_{i}{)}^{2}/\sum_{1}^{N}{\left(y_{i}\right)}^{2}\right)$$

(3) Mean absolute percentage error (MAPE)

Mean absolute percentage error represents the difference between the original and predicted values extracted by the averaged absolute difference over the dataset. It measures the prediction accuracy of the predictive model.

(9) $$MAPE=\frac{\sum_{i=1}^{N}((t_{i}-y_{i}{)/t}_{i})\times 100)}{N}$$

In the above equations, *t* is the target value, *y* is the predicted value, and *N* is the number of samples in the analysis.

## 4. Results and Discussions

### 4.1. Main Effects of Process Parameters on Cutting Force

**Figure 5:**
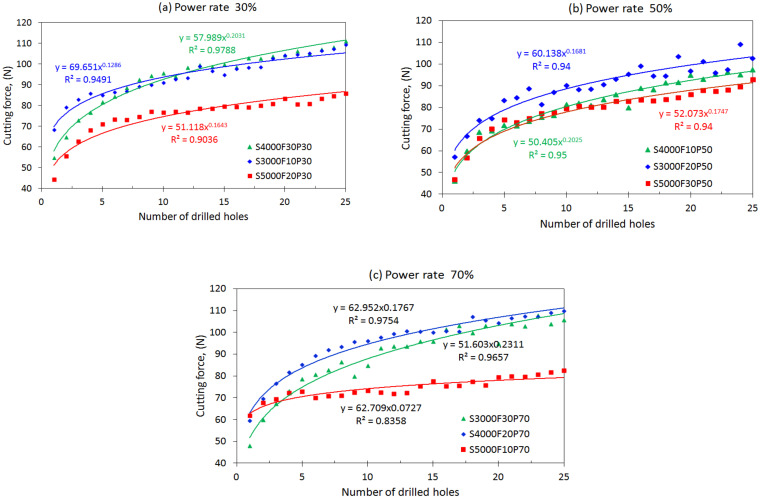
Evolutions of cutting force with the increase of drilling cycles under different process parameters.

Table 2 summarizes the cutting forces measured during each drilling process under various cutting conditions. The evolutions of the cutting force with increasing drilling cycles under different process parameters are illustrated in Figure 5. First, the analysis of variance (ANOVA) was conducted to observe the important level of the process parameters for the cutting force in the ultrasonic vibration-assisted drilling of CFRPs. The statistical coefficients from the ANOVA are presented in Table 3. As shown, the spindle speed contributes significantly to the thrust force, with a contribution percentage of 75.36%, followed by the feed rate and then the power rate of ultrasonic excitation, with contribution percentages of 5.30% and 4.52%, respectively. This implies that increasing the spindle speed can effectively reduce the cutting force. As shown in Figure 5, under ultrasonic power rates of 70% or 30%, a lower level of drilling force is generated at a high spindle speed of 5000 rpm as compared with other lower speeds.

**Figure 6:**
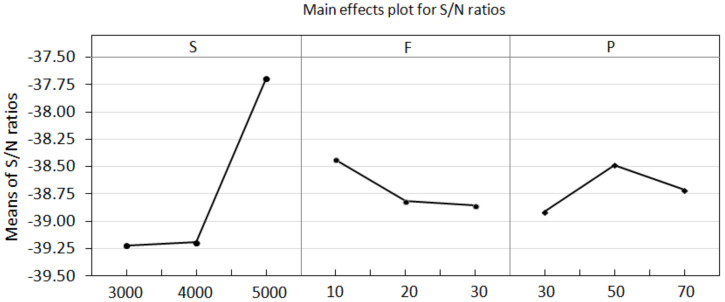
Main effects plot for cutting force.

**Figure 7:**
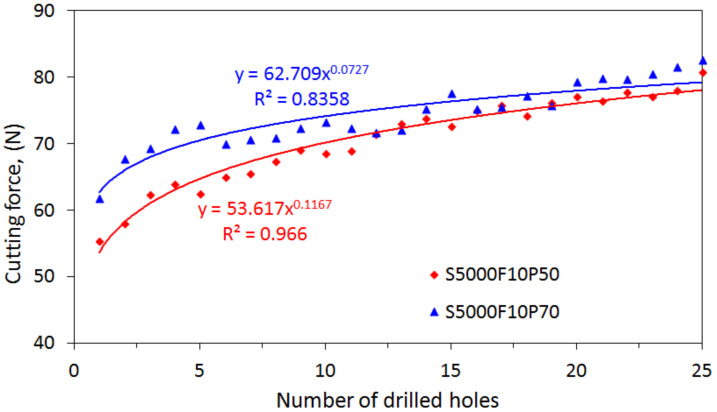
Comparisons of the cutting forces under process parameters A3B1C2 and A3B1C3.

Furthermore, based on the Taguchi method, the mean S/N ratios and mean values of the cutting force corresponding to different levels of each factor were calculated and are listed in Table 3. The main effect plots of these levels are used for evaluation, as shown in Figure 6. The S/N ratios change when the factor increases from a lower to a higher level. According to the principle of smaller-the-better machining, the corresponding levels of high S/N ratios are selected as the optimal machining conditions within the experimental ranges specified for this study. As shown in Figure 6, the cutting force is the minimum at the third spindle speed (A) level, the first feed rate (B) level, and the second power rate (C) level. Thus, the optimal combination of the process parameters for the minimum cutting force can be determined to be A3B1C2, with a spindle speed of 5000 rpm, a feed rate of 10 mm/min, a and power rate of 50%. Under these conditions, the mean value of the cutting force was calculated to be 72.5 N. To validate the optimum process parameter, another drilling test was conducted based on the optimal level of the process parameter using a new drill. The drilling was repeated 25 times under the same conditions. The measured cutting force with the increasing drilling cycles is illustrated in Figure 7. The mean value of the cutting force over the 25 drilling cycles is approximately 70.09 N, which is lower than the value (72.5 N) estimated by the Taguchi method. For comparison, another process parameter, A3B1C3, was selected by increasing the power rate to a higher level of 70%, with the spindle speed and feed rate kept at the same level. This yielded an estimated mean value of cutting force of 74.40 N, which agrees well with the measured mean value of 74.38%. For comparison, the evolution of the cutting force during drilling under the two sets of process parameters is shown in Figure 7, which clearly shows the effect of the process parameters on the cutting force during drilling. The result shows that the Taguchi method can be used to determine the optimum process parameters for drilling processes involving lower cutting forces.

The main effect analysis clearly indicates that the cutting forces are negatively correlated with the spindle speed and vibration amplitude and positively correlated with the feed rate. This phenomenon was also found in the study conducted by Cao et al. [37] using the UAD method, but, in a study of the conventional drilling of composites, Khashaba et al. [24] found that the feed rate was the most influential parameter affecting the drilling force. The influence of spindle speed is significant when the feed is at high levels. The effect of the vibration amplitude observed in this study was not as significant as that found in the study of Cao et al. [37], in which the ultrasonic amplitude was set at levels that were 5 to 17 μm higher than the UAD modules used in this study. Lower ultrasonic amplitude may not show a significant reduction in cutting force due to less ultrasonic energy against the cutting resistance [33].

### 4.2. Variations of Cutting Force with Drilling Cycles

As shown in Figure 5, the cutting force generated during drilling increased gradually with the number of drilled cycles. The increasing tendency or evolution of the cutting force is different for each tool operated under different process parameters. The cutting force at the initial drilling cycles is approximately 45 to 60 N, which then increases significantly to a higher value of approximately 90 N after 25 drilled holes. In fact, the variation in the cutting force with drilling cycles has been reported by Makhdum et al. [34], who mentioned that the variation is likely due to the wear of the cutting edge of the tool.

The relationship between the cutting forces (*F_t_*) and the number of drilled holes (N) can be fitted appropriately using the power-law formula, $\;F_{t}=\alpha N^{\beta }$, where α and β are coefficients that depend on the combination of process parameters used for a specific tool. For example, as shown in Figure 5b, for drilling under an ultrasonic power rate of 50%, a spindle speed of 3000 rpm, and a feed rate of 20 mm/min, the cutting force can be expressed in terms of the drilling cycles by the formula $\;F_{t}=60.138N^{0.1681}$, with the correlation coefficient R = 0.94. The cutting force is $F_{t}=52.073N^{0.1747}$ at a speed of 5000 rpm, a feed rate of 30 mm/min, and a power rate of 50%. Similarly, at power rates of 30% and 70%, the cutting force increases at different rates with the increase in the number of drilled holes, which is governed significantly by the process parameters, i.e., the spindle speed and feed rate. As shown in Figure 5c, a significantly lower level of cutting force is generated when drilling at a spindle speed of 5000 rpm, a feed rate of 10 mm/min, and a power rate of 70% as compared with other conditions. Furthermore, at a power rate of 30%, a lower cutting force is generated in the drilling at higher spindle speeds, such as 5000 rpm, than at lower speeds.

**Figure 8:**
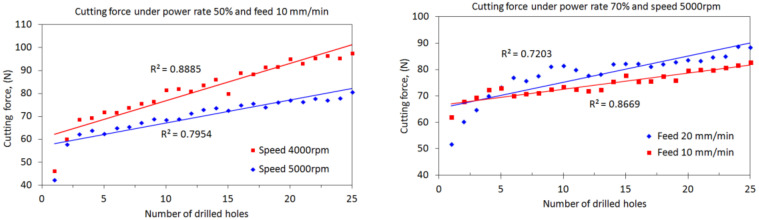
Effects of spindle speed and feed rate on the cutting forces.

The comparisons of the results presented in Figure 5 clearly indicate that, at specific a power rate, the cutting speed will dominate the cutting force, followed by the feed rate. A higher speed with a lower feed rate induces a lower cutting force as compared with a lower speed with a higher feed rate. Further, it can be observed from Figure 8 that, at the same power rate and feed rate, a lower cutting force can be achieved in a higher cutting speed as compared with a low speed. The difference in cutting force between the two cases will gradually increase with drilling cycles. This again reveals the dependence of the cutting force on the cutting speed. Similarly, at the same power rate and spindle speed, lower forces will be generated under low feeding drilling as compared with drilling under a high feed rate. However, the effect of the feed rate on the evolution of cutting force with drilling cycles is not significant.

In practice, the cutting force is one of the main factors affecting tool wear. In fact, this phenomenon has been observed in drilling experiments involving the RAD process. The wear on the cutting edge of the drill may further increase the cutting resistance and hence the cutting force. The current results clearly show that the appropriate process parameters must be selected to reduce the cutting force.

In this study, the results obtained from drilling experiments clearly indicate the evolution scenarios of the cutting force with increasing drilling holes under specific process conditions. Similar phenomena were also reported in prior studies [34,44]. Makhdum et al. [34] showed that the evolution of cutting force with drilling cycles was apparently observed, but there was no significant difference in increasing tendency under a feed rate at 8 mm/min and 16 mm/min, respectively. Azghandi et al. [44] also found that cutting force increased drastically at the initial stage due to the tool wear in drilling and slightly began to decrease with the increase in ultrasonic vibration amplitude. Their results verified that UAD can reduce the drilling forces during certain feed rates, and the effects of ultrasonic vibration will weaken when the feed rate exceeds an optimum threshold. This may imply the interactive effect of the process parameters, which are similar to the phenomena observed in this study—that is, the evolution rate of cutting forces was affected to change in different trends by the process parameters used in the drilling process. This means that the influence extent of the spindle speed and feed rate on the cutting force may vary with the drilling cycles. This is probably due to the wearing of the tool with time, but this needs to be clarified in future studies.

### 4.3. Quantification of the Delamination Factor

**Figure 9:**
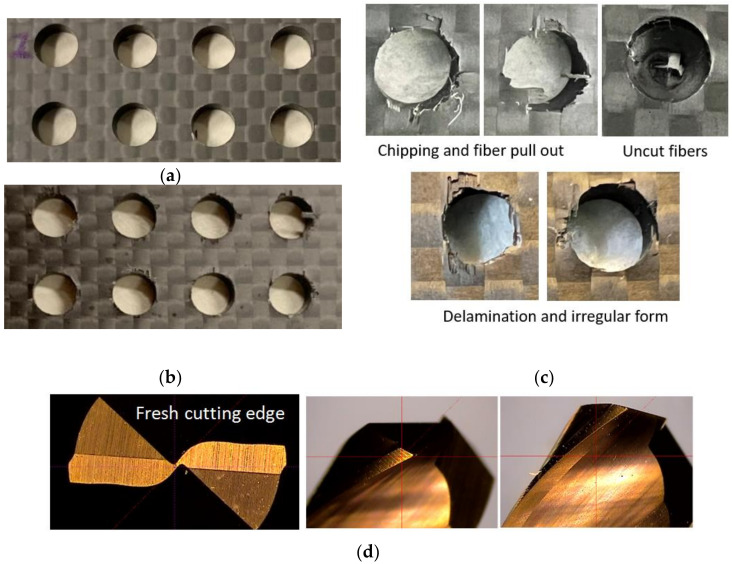

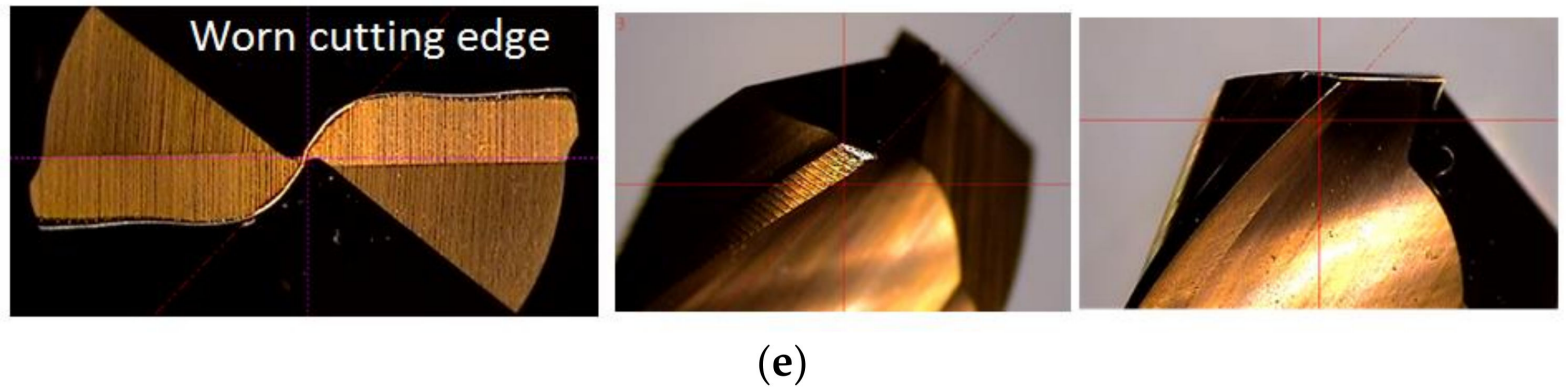
Photos of drilled CRFP laminates and microscopic graphs of the flank edge of a tool. (**a**) Entry surface of drilled holes, (**b**) exit surface of drilled holes with delamination and peripheral burrs, (**c**) damage patterns, (**d**) fresh cutting edge, (**e**) worn cutting edge after 25 holes.

**Figure 10:**
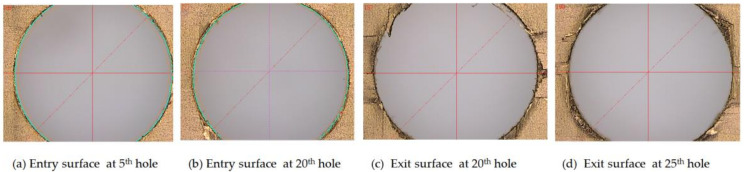
Typical surface morphologies of the drilled CRFP laminate.

**Figure 11:**
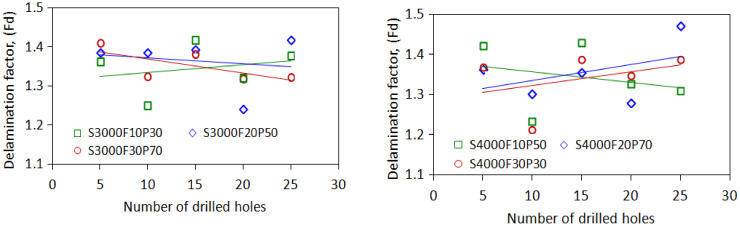

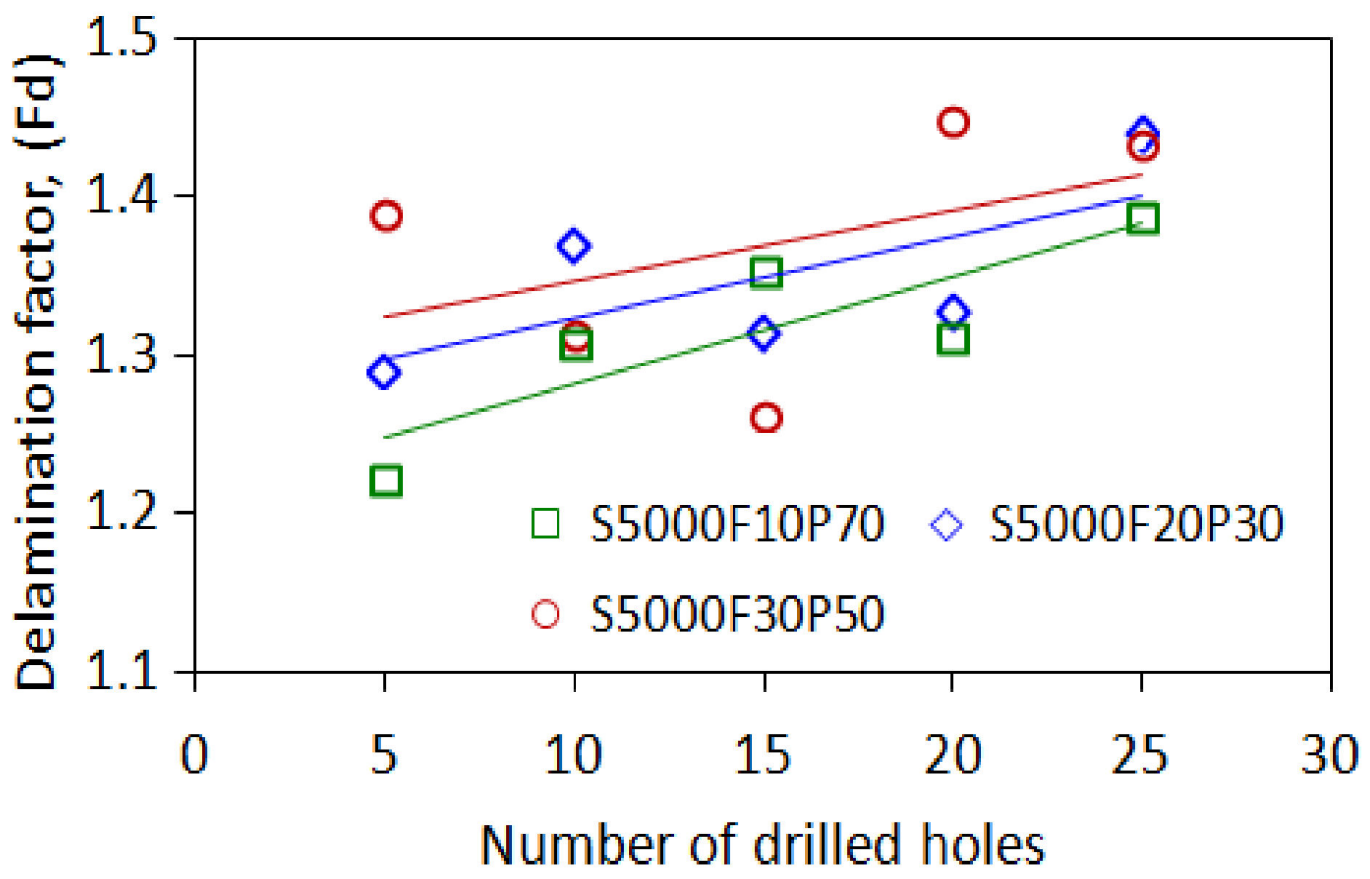
Variations in the delamination factor with drilling cycles.

**Figure 12:**
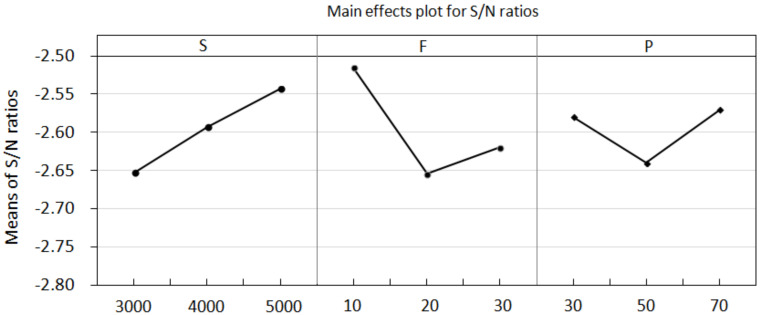
Main effects plot for the delamination factor.

Figure 9 presents photographs of drilled CRFP laminates and microscopic graphs of the flank edge of a tool, which show significant differences in the drilling results obtained using the tool with a fresh edge and a worn-out edge, separately. As shown in Figure 9a, the holes machined using the new tool with a fresh cutting edge exhibit a smooth inner surface and high integrity. Meanwhile, as shown in Figure 9b, the holes drilled using the tool with a worn cutting edge show surface chipping, fuzzing, and uncut fibers. Furthermore, the cutting edge of the drill wore out after 80 drilling cycles. The surface delamination of the drilled holes is illustrated in Figure 10. The delamination factors were measured at the exit surfaces of the drilled holes, as presented in Table 4. The variations in the delamination factor with the number of drilling cycles are illustrated in Figure 11. The effects of the process parameters on delamination were evaluated using the Taguchi method and ANOVA. The results of the ANOVA and S/N ratio of the delamination factor for the CFRP laminate are presented in Table 5. As shown, the delamination factor affects the feed rate the most significantly, with a contribution percentage of 46.57%, followed by the spindle speed and power rate of ultrasonic excitation, with contribution percentages of 26.64% and 12.54%, respectively. This indicates that the feed rate exerts a more significant effect on the drilling quality than the other parameters. The mean S/N ratios of the delamination factor at different levels for each process parameter were calculated based on the Taguchi method. Based on the machining quality requirement for drilled holes, the smaller-the-better principle was applied to evaluate the effects of the process parameters on the delamination factor. The main effect plots for these levels are shown in Figure 12. As shown in the figure, the optimal process parameters within the designated ranges for minimum delamination are those of A3B1C3, i.e., a spindle speed of level 3 (5000 rpm), a feed rate of level 1 (10 mm/min), and a power rate of level 3 (70%). This implies that a lower feed speed and a higher cutting speed can reduce the surface delamination of the drilled hole, which is correlated with the wear of the cutting edge with the number of drilling cycles [43].

Regarding the dependence of the delamination factor on the evolution of the cutting force, the delamination does not increase significantly with the drilling cycles of the cutter in some cases, although the cutting forces increase with the number of drilling cycles of the cutter. However, in other cases under specific cutting conditions, such as S5000F10P70 and S5000F20P30, the delamination factor increases with the number of drilled holes, with R = 0.69–0.85.

The delamination of the drilled hole is sensitive to the cutting force and flank wear of the drill, which have been experimentally verified to increase with the number of drilling cycles [39,45,46]. The current results show that, in the drilling cases (S5000F10P70 and S5000F20P30), the cutting forces increase significantly with the number of drilling cycles, where positive correlations are indicated with the delamination factor (R = 0.69–0.85). Typically, the surface damage and material delamination of CFRPs are generated by the drilling force exerted on the laminate material, which can be affected by many factors such as material composition, laminate layers, drill geometry, and cutting conditions [7,36]. Delamination can be evaluated by measuring the damaged zone in terms of area or diameter using different methods. However, the measurement of the damaged area is questionable because the delamination is caused by only a few fibers peeled up around the hole periphery [39,45,47]. This may cause an inaccurate quantification of the delamination factor for reflecting the extent of substantial damage and hence questionable quantifications of the delamination factor in some drilling cases. To observe the variation in the delamination factor with the increasing number of drilling cycles, more drilling tests should be conducted to further investigate the effects of process parameters in long-term drilling operations.

### 4.4. Regression Models for Cutting Force

The independent variables used in the regression analysis included the spindle speed (*S*), feed rate (*F*), ultrasonic power rate (*P*), and number of drilled holes (*N*). Datasets were obtained from machining experiments under different process parameters, where 225 records pertain to the drilling process under nine different combinations of machining parameters. The regression coefficients for the cutting force models are listed in Table 6 and Table 7. The mathematical models are expressed in the forms of nonlinear polynomial and power-law functions.

(10) $$F_{t}=4.7497+1.3836\times N-0.3663\times F+0.0418\times S+0.0345\times P-0.0158\times F^{2}-6.84\times 10^{-6}\times S^{2}+3.04\times 10^{-4}\times F\times S$$

(11) $$F_{t}=840.773\times F^{0.0392}\times S^{-0.3249}\times P^{-0.0288}\times N^{0.1682}$$

**Figure 13:**
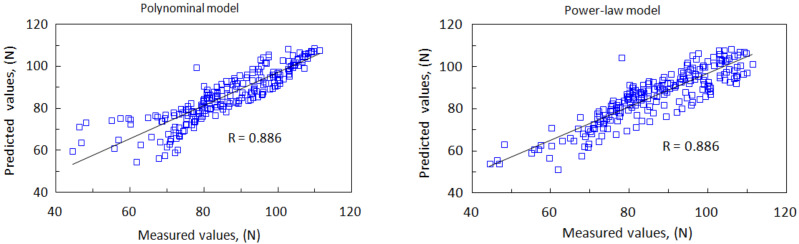
Comparisons of the cutting force between measurements and predictions.

Based on the regression coefficients, the *p*-value of the spindle speed and its interactions with the feed rate against the cutting force are significantly less than 0.05, indicating that the spindle speed exerts a more significant effect on the cutting force. As illustrated in Figure 5, drilling at a higher cutting speed induces lower cutting forces. The statistical values of the two regression models, such as the R, RMSE, and MAPE, were used as the basis to determine the prediction performance, as summarized in Table 8. As shown in Figure 13, the predicted cutting forces agree well with the measurements. The coefficient of determination (R) is approximately 0.886.

Furthermore, the mean absolute percentage error for the nonlinear polynomial model is approximately 6.52%, with an RMSE of 6.43. For the power-law model, the average percentage of prediction error is approximately 6.37%, with an RMSE of 5.94. This clearly indicates that both the nonlinear polynomial and power-law models can accurately predict the cutting forces during the drilling of CFRPs under different machining conditions. As demonstrated in Section 4.1, under specific cutting conditions, the cutting force can be fitted using a power-law model with a higher R.

### 4.5. Validation of the Models

To validate the prediction performance of the cutting force models, additional drilling tests were conducted under specific conditions based on the same machining configuration used in the previous tests. The cutting conditions were specified at levels different from those adopted in previous experiments.

(1) S = 3000 rpm; F = 20 mm/min; *p* = 70%
(2) S = 4000 rpm; F = 30 mm/min; *p* = 70%
(3) S = 5000 rpm; F = 10 mm/min; *p* = 50%
(4) S = 5000 rpm; F = 20 mm/min; *p* = 70%

**Figure 14:**
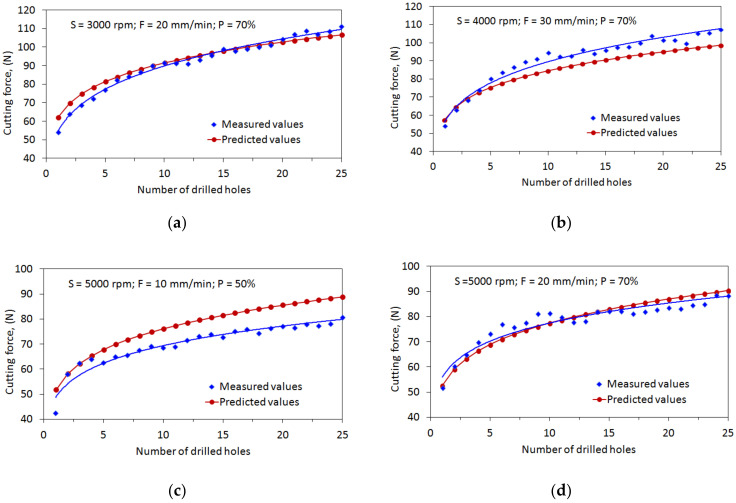
Comparisons of the evolutions of cutting force with drilling cycles (validation cases). (**a**) S = 3000 rpm; F = 20 mm/min; *p* = 70%, (**b**) S = 4000 rpm; F = 30 mm/min; *p* = 70%, (**c**) S = 5000 rpm; F = 10 mm/min; *p* = 50%, (**d**) S = 5000 rpm; F = 20 mm/min; *p* = 70%.

Four new tools were used for drilling under specific cutting parameters. For each tool, the cutting forces in each drilling cycle were measured using a dynamometer, as shown in Figure 14. The cutting forces for the four drilling cases were predicted using the individual power-law function and illustrated in the Figure 13 comparisons.

It is noted that, in first case (S3000 rpm; F20 mm/min; P70%), the cutting forces predicted by the power-law model show a similar evolution trend to that observed in the drilling experiments. The RMSE and MAPE between predictions and measurements are approximately 3.41 and 3.38%. The measured forces can be well correlated with the number of drilled holes by the equation $F_{t}=56.178N^{0.2023}$, with R^2^ = 0.97, while the predictive model for this drilling case can be obtained as $F_{t}=62.053N^{0.1682}.$

The prediction performances for four validation cases were verified in terms of the R, RMSE, and MAPE, as listed in Table 8. For the polynomial model, the RMSE and MAPE of the four validation cases are approximately 5.63 and 5.52%, respectively. For the power-law model, the RMSE and MAPE are 5.63 and 5.52%, respectively. The validation tests clearly demonstrate that the regression models can be considered as appropriate tools for predicting the cutting force in the ultrasonic vibration-assisted drilling of CFRPs.

### 4.6. Control of Cutting Force

**Figure 15:**
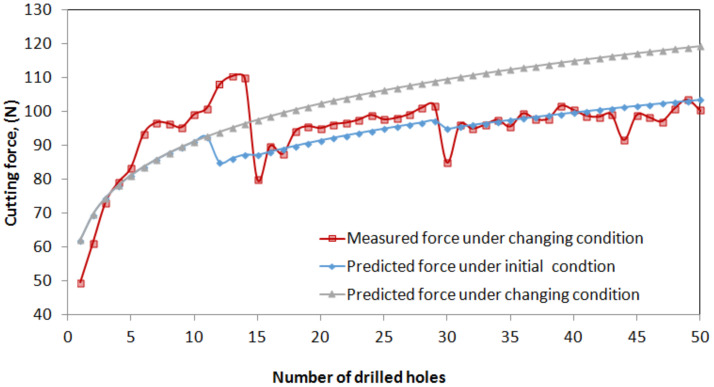
Comparisons of the measured cutting force during the drilling process and the predicted values.

A predictive model of the cutting force was successfully established and verified to demonstrate its superior prediction performance. The predictive model is expected to predict the variation in the cutting force during drilling under various cutting conditions. To prevent severe damage to the tool or workpiece during drilling, the cutting force should be reduced by appropriately selecting or adjusting the cutting conditions for the drilling process, e.g., by increasing the spindle speed or the power rate of ultrasonic vibration excitation. To demonstrate the feasibility of this concept, a series of drilling tests were conducted using the same experimental setup. A new tool was used to drill holes for up to 50 cycles under various cutting conditions such that the cutting force was suppressed to a lower value when it was measured at a higher level, e.g., 110 N as the threshold of the cutting force. The experimental process associated with the adjustment of the cutting conditions is described as follows. For comparison, the measured cutting force during the drilling process and the values predicted by the power-law model are illustrated in Figure 15.

1. During initial machining (holes #1–#11), a conservative cutting condition was selected, i.e., a speed of 3000 rpm, a feed rate of 10 mm/min, and a power rate 30%. The cutting force measured for drilled holes #1 to #11 increased from 49 to 100 N, which is similar to the results of previous tests. The predicted values ranged between 60 and 90 N, i.e., lower than the measured values.
2. In the second stage (holes #12–#14), to reduce the cutting force, a cutting condition with higher values was selected, i.e., a speed of 4000 rpm, a feed rate of 20 mm/min, and a power rate 70%, with a predicted force of 85–87 N. However, the measured cutting forces were higher, i.e., 108–110 N.
3. In the third stage (holes #15–#29), a lower feed rate (10 mm/min) and power rate (50%) were selected for the drilling process at a speed of 4000 rpm. Consequently, the measured cutting force decreased immediately to 80 N but increased gradually from 89 to 101 N during the subsequent process.
4. In the fourth stage (holes #30–#50), a higher spindle speed (5000 rpm), feed rate (30 mm/min), and power rate (50%) were selected. At hole #30, the cutting force decreased to a lower value of 85 N; however, it increased gradually to 103 N in the subsequent process from holes #31–#50.

Based on Figure 15, under the initial process parameters without adjustment, the cutting force is predicted to increase to 120 N in the subsequent drilling cycles around hole #50, at which point severe damage to the drills or material is expected to occur. However, the cutting force was controlled below the desired value of approximately 100 N by selecting the appropriate cutting conditions timely, which can be determined in advance using the regression model. As shown in the figure, the predicted cutting forces agree well with the measured forces, with RMSE and MAPE values of 7.55 and 5.61%, respectively. This verifies the feasibility of the predictive model for predicting the variation in cutting force during drilling processes and provides suggestions for the adjustment of process parameters to suppress the cutting force below the desired value.

## 5. Conclusions

In this study, the variation in cutting force during the drilling of CFRP materials using an ultrasonic vibration-assisted drilling system was investigated. The effects of the process parameters on the cutting force were analyzed. Based on the results of comprehensive machining tests, the following conclusions were obtained:

1. Based on the Taguchi method and ANOVA, the spindle speed showed a significant effect on the cutting force, followed by the feed rate and the ultrasonic power rate, respectively. Additionally, the most significant parameter affecting the delamination factor was the feed rate, followed by the spindle speed and the ultrasonic power rate.
2. The evolution scenarios of the cutting force with the influence of process parameters were observed in the drilling of CFRPs, which can be affected to different increasing tendencies by the process parameters. Additionally, the increasing tendency of the cutting force with drilling cycles has been successfully predicted by using the proposed regression models with superior prediction performance.
3. The predictive power-law model was demonstrated to be an effective basis for selecting the drilling parameters to suppress the cutting force on CFRP composites during continuing drilling cycles.
4. The delamination damage of CFRP laminates and its dependence on the cutting force and number of drilling cycles, which are important for controlling the machining quality, should be further investigated through experiments.

## Figures and Tables

**Table 1:** Experiment parameters and their levels.

Process Parameter	Notation	Level		
		1	2	3
Spindle speed (S, rpm)	A	3000	4000	5000
Feed rate, (F, mm/min)	B	10	20	30
Ultrasonic power rate, (P, %)	C	30	50	70

**Table 2:** Cutting forces measured under different process parameters.

	L1	L2	L3	L4	L5	L6	L7	L8	L9
Speed (rpm)	3000	3000	3000	4000	4000	4000	5000	5000	5000
Feed (mm/min)	10	20	30	10	20	30	10	20	30
Power rate (%)	30	50	70	50	70	30	70	30	50
Drilled cycle	Cutting force (N)								
1	68.51	57.34	48.10	46.25	59.62	55.06	61.87	44.48	46.86
2	79.34	66.94	60.06	60.08	69.65	64.97	67.86	55.81	56.90
3	83.04	74.19	67.51	68.75	76.60	73.20	69.37	62.88	65.78
4	85.99	75.09	73.27	69.48	81.64	76.99	72.25	68.20	70.32
5	85.13	83.41	78.72	71.91	85.19	81.86	72.94	71.09	74.37
6	86.67	84.59	80.88	71.78	89.26	84.6	70.03	73.54	73.21
7	87.00	88.79	82.88	73.79	92.04	88.81	70.77	73.34	75.06
8	89.44	81.53	86.46	75.71	93.39	92.67	70.98	74.66	77.38
9	90.23	87.19	80.13	76.58	95.67	94.54	72.47	77.24	77.72
10	91.15	90.34	85.43	81.44	96.00	95.89	73.31	76.70	79.60
11	92.83	88.46	92.89	82.10	97.79	94.24	72.43	77.19	80.57
12	93.45	88.68	93.64	81.04	99.41	98.66	71.78	76.73	80.26
13	99.08	90.66	93.73	83.74	100.52	99.79	72.22	78.71	80.27
14	96.81	93.22	95.98	86.19	100.49	99.01	75.32	78.56	82.90
15	94.92	95.42	95.99	80.00	99.96	100.03	77.71	79.65	82.95
16	97.81	99.20	101.58	88.96	100.57	98.85	75.35	79.47	83.58
17	98.59	94.59	103.18	88.40	100.33	103.05	75.61	79.24	83.06
18	98.71	94.74	99.96	91.52	107.20	102.96	77.35	80.06	83.69
19	102.82	103.53	103.15	91.73	105.50	104.04	75.85	80.93	84.67
20	104.37	96.92	95.25	95.16	104.23	104.61	79.49	83.51	86.12
21	104.75	101.36	104.04	93.13	106.56	106.56	79.90	80.67	87.93
22	105.38	96.10	103.00	95.48	107.40	105.26	79.77	80.86	87.51
23	106.61	97.60	108.00	96.55	107.08	107.39	80.68	83.40	88.22
24	107.46	109.24	104.01	95.38	108.99	108.33	81.64	84.73	89.67
25	109.66	102.80	105.81	97.58	109.95	111.38	82.66	86.06	93.06

**Table 3:** ANOVA coefficients and mean S/N ratios.

Process Parameter	Level 1	Level 2	Level 3	Degree of Freedom (DF)	Sum of Square (SS)	Mean of Square (MS)	*F*-Value	Contributions
A Spindle speed, (S)	−39.22	−39.19	−37.69	2	4.58606	2.2930	5.0879	75.36%
B Feed rate, (F)	−38.44	−38.82	−38.86	2	0.32274	0.16136	0.3580	5.30%
C Power rate, (P)	−38.91	−38.48	−38.71	2	0.27522	0.13761	0.3053	4.52%
Error, (e)				2	0.90136	0.45068		14.81%
Total				8	6.08537	3.04268		

**Table 4:** Delamination factors measured for specific drilled holes for nine drilling sets.

Drilling Set	Spindle Speed (rpm)	Feed Rate (mm/min)	Power Rate (%)	Delamination Factor Measured at Specified Drilled Holes					
				Y1 (#5)	Y2 (#10)	Y3 (#15)	Y4 (#20)	Y5 (#25)	S/N Ratio
1	3000	10	30	1.361	1.248	1.417	1.320	1.377	−2.579
2	3000	20	50	1.385	1.385	1.393	1.241	1.418	−2.708
3	3000	30	70	1.411	1.324	1.381	1.318	1.322	−2.672
4	4000	10	50	1.421	1.232	1.430	1.326	1.308	−2.576
5	4000	20	70	1.363	1.303	1.356	1.280	1.473	−2.649
6	4000	30	30	1.367	1.212	1.388	1.347	1.386	−2.553
7	5000	10	70	1.220	1.306	1.353	1.310	1.388	−2.389
8	5000	20	30	1.290	1.370	1.315	1.328	1.441	−2.606
9	5000	30	50	1.388	1.311	1.262	1.448	1.432	−2.634

**Table 5:** Response table for the S/N ratios and ANOVA of the delamination factor.

Control Factors		Level 1	Level 2	Level 3	Degree of Freedom (DF)	Sum of Square (SS)	Mean of Square (MS)	*F*-Value	Contributions (P%)
A	Spindle speed, (S)	−2.653	−2.593	−2.543	2	0.0181	0.0090	5.0879	26.64
B	Feed rate, (F)	−2.515	−2.654	−2.620	2	0.0316	0.0158	0.3581	46.57
C	Power rate, (P)	−2.579	−2.639	−2.570	2	0.0085	0.0043	0.3053	12.54
Error					2	0.0097	0.0048		14.25
Total					8	0.0679	0.0340		

**Table 6:** Regression coefficients for the cutting force model in nonlinear polynomial function.

Parameters	Coefficients	Standard Deviations	*p*-Value
Intercept	4.7497	15.8287	0.7644
Drilled cycles (*N*)	1.3836	0.0605	0.0000
Feed rate (*F*)	−0.3663	0.4997	0.4643
Spindle speed (*S*)	0.0418	0.0076	0.0000
Power rate (*P*)	0.0345	0.0338	0.3082
Feed (*F*^2^)	−0.0158	0.0093	0.0891
Speed × speed (*S*^2^)	−6.84 × 10^−6^	0.0000	0.0000
Speed × feed rate (*F* × *S*)	0.0003	0.0001	0.0004

**Table 7:** Regression coefficients for the cutting force model in power-law function.

Parameters	Coefficients	Standard Deviations	*p*-Value
Intercept	6.7343	0.2189	0.0000
Spindle speed (*S*)	−0.3249	0.0251	0.0000
Feed rate (*F*)	0.0392	0.0116	8.52 × 10^−4^
Power rate (*P*)	−0.0288	0.0151	5.70 × 10^−2^
Drilled cycle (*N*)	0.1682	0.0064	0.0000

**Table 8:** Prediction performance of regression models.

Model Type	Regression Training Dataset			Validation Dataset		
	R	RMSE	MAPE	R	RMSE	MAPE
(a) Polynomial model	0.886	6.43	6.52%	0.925	5.63	5.52%
(b) Power-law model	0.886	5.94	6.37%	0.934	5.47	5.86%
